# A high plasma D-dimer level predicts poor prognosis in gynecological tumors in East Asia area: a systematic review and meta-analysis

**DOI:** 10.18632/oncotarget.17936

**Published:** 2017-05-16

**Authors:** Lei Xu, Fan He, Hongcai Wang, Bei Gao, Huini Wu, Shuping Zhao

**Affiliations:** ^1^ Gynecology Department, Qingdao University Affiliated Qingdao Women's and Children's Hospital, Qingdao, China; ^2^ Gynecology Department, Maternal and Child Health Hospital of Zibo City, Shandong, China; ^3^ Department of Molecular Oncology, H. Lee Moffitt Cancer Center & Research Institute, Tampa, FL, USA; ^4^ State Key Laboratory of Ophthalmology, Zhongshan Ophthalmic Center, Sun Yat-sen University, Guangzhou, China; ^5^ Department of Biological Sciences, University of Illinois at Chicago, Chicago, IL, USA

**Keywords:** D-dimer, gynecological tumors, meta-analysis, prognosis

## Abstract

High pre-treatment plasma D-dimer levels have been reported as a factor associated with a poor prognosis in different types of malignancies, including pancreatic, gastric, colorectal, lung, and nasopharyngeal carcinoma. Here, we performed a meta-analysis to determine the association of plasma D-dimer levels and long term survival in gynecological cancers, including ovarian, cervical and endometrial carcinoma. We searched all eligible publications in PubMed and Web of Science Databases up to August 2016. Primary outcomes, including overall survival (OS), disease-free survival and hazard ratios (HR) of were extracted and analyzed. Heterogeneity and publication bias were also assessed. A total of 7 eligible studies with 1112 cases were included in this study and all included studies are conducted in East Asia area. We found that gynecological cancer patients with high D-dimer demonstrates a much lower 5-year survival rate than those with low D-dimer levels (OR 4.12, 95% CI 3.04-5.58, P<0.00001). No significant heterogeneity is found (I^2^ = 10 %; P = 0.35). Importantly, pooled analysis showed that high plasma D-dimer levels are predictive of a shorter OS in gynecological cancers (HR 2.09, 95% CI 1.59-2.74). No heterogeneity is observed (I^2^=5%, P=0.39). Additionally, a subgroup analysis of ovarian cancer is conducted. In conclusion, this meta-analysis showed that a high plasma D-dimer level predicts poor prognosis in gynecological tumors.

## INTRODUCTION

Gynecological cancers, including ovarian, endometrial and cervical cancers, are major types of malignancies of genital system for women worldwide. In the United States, 95000 women are estimated to be diagnosed with gynecological cancers and 28800 will die of it in 2016 [1]. According to the GLOBOCAN database, the incidence and mortality of gynecological cancers are lower in East Asia than North America area. However, because of the huge population in this area, it accounts for more than 20% of the incidence worldwide. There are about 750,000 new gynecological carcinoma cases worldwide and about 150,000 of them are from East Asia in 2012(20%). Targeted therapeutics, such as EGFR inhibitors and other Serine/Threonine kinase inhibitors have shown limited efficacy in gynecological cancers and current therapies remain to be radical surgical tumor debulking plus platinum-based chemotherapy [2]. Thus, effective prognostic biomarkers are in great need to distinguish gynecological cancer patients who require more aggressive treatments.

Activation of coagulation and fibrinolysis have been frequently observed in many tumors [3]. Coagulation refers to the process of changing blood from liquid to gel, forming clots. Fibrinolysis is the degradation of fibrin to prevent blood clots from forming. D-dimer is two cross-linked D fragments of fibrin protein, the product of fibrin degradation [4]. In gynecological cancers, both coagulation and fibrinolysis systems are hyperactivated. Thus, the level of plasm D-dimer is also found elevated and associated with the formation of venous thromboembolism (VTE) [5]. Recently, elevated levels of D-dimer have shown predictive of survival in many malignancies, including lung, pancreatic, colorectal and breast cancer [6–9]. The relationship between high plasma D-dimer level and poor prognosis is also reported in gynecological cancers, including ovarian, cervical and endometrial cancers [10–16]. However, a systematic study is needed to address and to confirm the significance. The aim of this meta-analysis is to evaluate the prognostic significance of D-dimer across gynecological cancers, thereby providing references for clinical decisions.

## RESULTS

### Literature search

**Figure 1:**
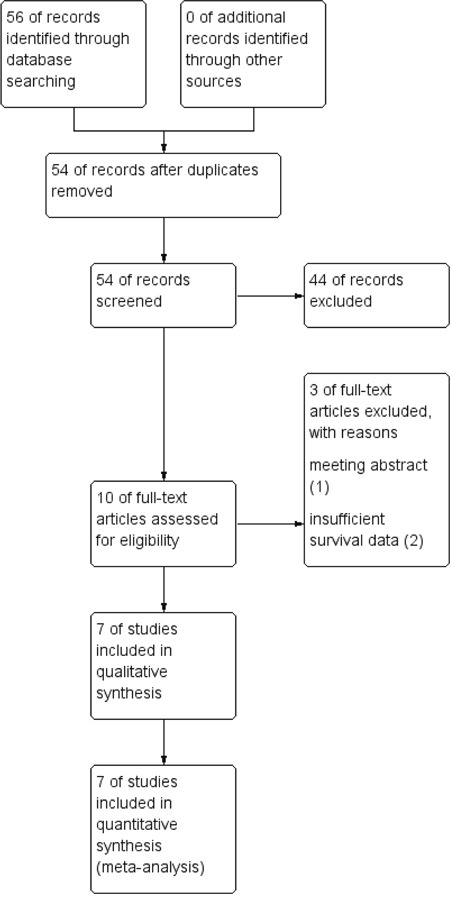
Flow diagram of search strategy in the meta-analysis

A total of 56 publications were identified in the initial literature search. Based on screening of titles or abstracts, 46 records were excluded. Full text articles were retrieved only for 10 publications and assessed for eligibility. 3 publications were excluded for insufficient data. Finally, 7 studies were included in this meta-analysis [10–16]. The process of identifying studies is shown as a flow chart in Figure 1. A total of 1112 patients were included and the characteristics of seven eligible studies are summarized in Tables 1.

**Table 1:** Main characteristics of seven included studies in this meta-analysis

Study	Year	Tumor type	Patient source	patient number	Multivariate/univariate	HR(95%)	P value	60 months OS rates
Sakurai	2015	Ovarian	Japan	134	Multivariate	2.11 (1.02-4.78)	0.041	61.60%
Man	2015	Ovarian	China	190	Multivariate	1.643(1.025-2.634)	0.039	53.68%
Liu	2015	Ovarian	China	125	Multivariate	1.901 (1.021-3.540)	0.043	53.60%
Nakamura	2016	Cervical	Japan	129	Multivariate	2.33(1.121-5.504)	0.043	73.64%
Luo	2015	Cervical	China	296	Multivariate	2.148(1.035-4.457)	0.036	67.9%
Nakamura	2016	Endometrial	Japan	110	Multivariate	19.646(1.874-206.011)	0.013	83.33%
Li	2015	Endometrial	China	282	Multivariate	3.968 (1.495–10.528)	<0.05	86.50%

### Meta-analysis results

**Figure 2:**
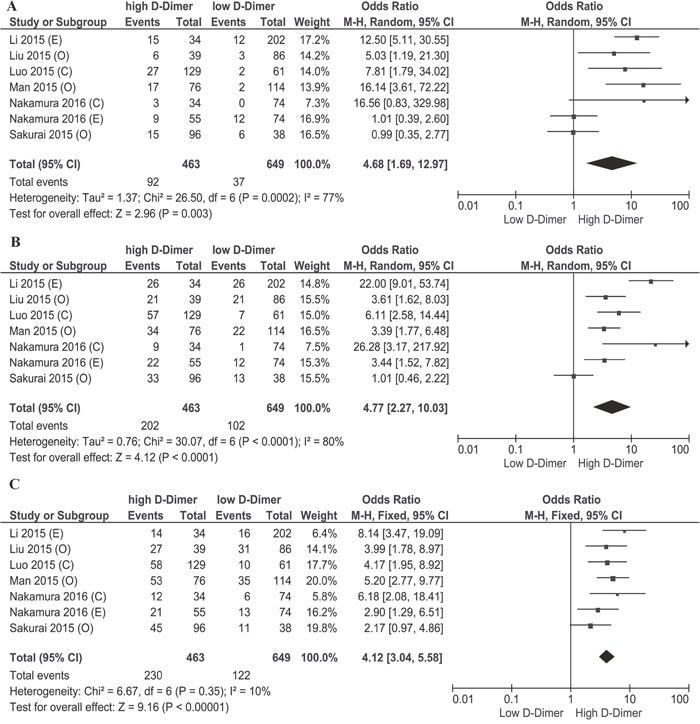
Forest plot of overall survival (OS) rates of high and low plasma D-dimer **(A)** Forest plot of 1-year OS rates of high and low plasma D-dimer. **(B)** Forest plot of 3-year OS rates of high and low plasma D-dimer. **(C)** Forest plot of 5-year OS rates of high and low plasma D-dimer. C: cervical cancer, O: ovarian cancer, E: endometrial cancer.

**Figure 3:**
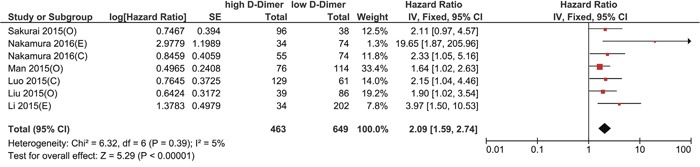
Forest plot of hazard ratio for the association of high plasma D-dimer level and OS; C: cervical cancer, O: ovarian cancer, E: endometrial cancer

**Figure 4:**
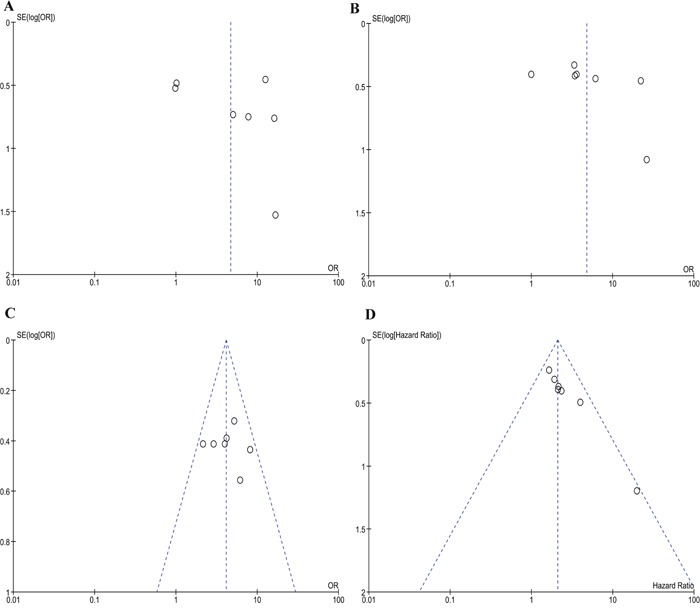
Begg's funnel plots of publication bias **(A)** Meta-analysis of 1 year overall survival rates of high and low plasma D-dimer. **(B)** Meta-analysis of 3 year overall survival rates of high and low plasma D-dimer. **(C)** Meta-analysis of 5 year overall survival rates of high and low plasma D-dimer. **(D)** Meta-analysis of hazard ratio for the association of high plasma D-dimer level and OS.

Based on the Kaplan-Meier survival curves of these seven studies, we extracted and calculated 1-year, 3-year, and 5-year OS data. The estimated proportion of heterogeneity (I^2^) between these seven studies was 10% (P = 0.35) for 5-year OS rates. Therefore, no significant heterogeneity exists and a fixed-effect model was applied. However, heterogeneity exists in 1-year and 3-year OS rates and a random-effect model was applied. As Figure 2 demonstrates, patients with a high plasma D-dimer level had lower 1-year, 3-year, and 5-year OS rates than patients with a low plasma D-dimer level (80.13% [371/463] vs 94.30% [612/649]; 56.30% [261/463] vs 84.28% [547/649]; 50.3% [233/463] vs 81.2% [527/649]). OR and 95% CI were all larger than 1, which supports that high plasma D-dimer level is a risk factor for patients. To further evaluate the prognostic effect of plasma D-dimer level in gynecological cancers, we used the multivariate HRs and their 95% CI in these studies to calculate a combined HR, demonstrating that patients with a high plasma D-dimer level had a worse prognosis than patients with a low plasma D-dimer level (HR 2.09, 95% CI 1.59-2.74, Figure 3). No heterogeneity was observed (I^2^=5%, P=0.39). Begg's funnel test was performed to estimate the publication bias of the literatures in this study. As shown in Figure 4, the shape of the Funnel plots showed no obvious evidence of asymmetry.

**Figure 5:**
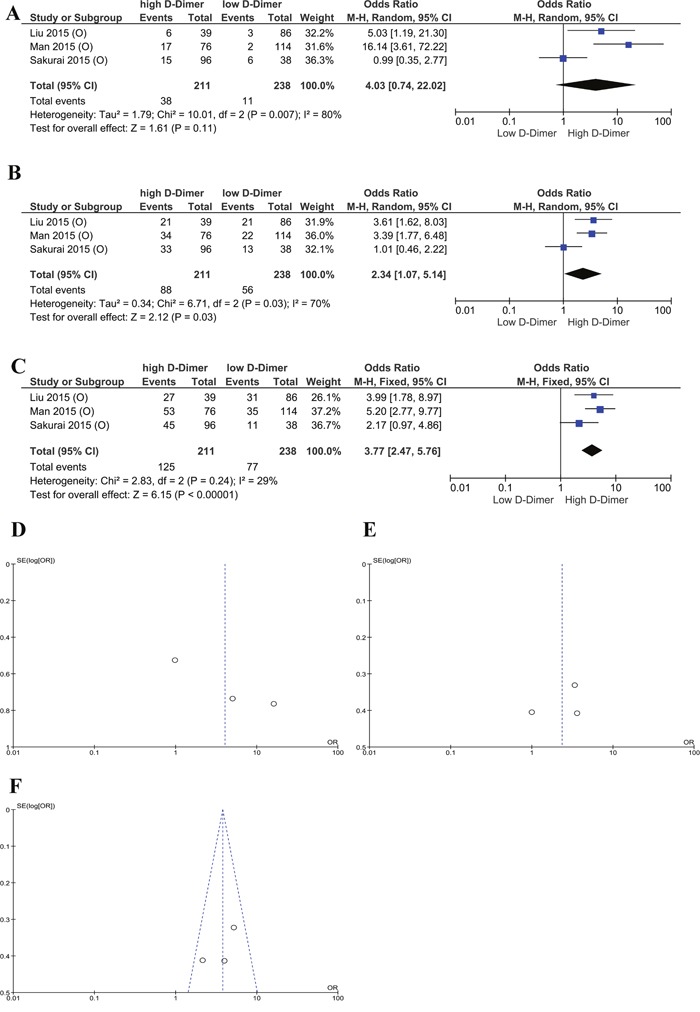
Subgroup analysis of ovarian cancer **(A)** Forest plot of 1 year OS rates of high and low plasma D-dimer. **(B)** Forest plot of 3-year OS rates of high and low plasma D-dimer. **(C)** Forest plot of 5-year OS rates of high and low plasma D-dimer. **(D)** Begg's funnel plots of publication bias of 1-year OS rates of high and low plasma D-dimer. **(E)** Begg's funnel plots of publication bias of 3-year OS rates of high and low plasma D-dimer. **(F)** Begg's funnel plots of publication bias of 5-year OS rates of high and low plasma D-dimer.

In addition, we understand that tumor development, treatment and prognosis among ovarian, cervical and endometrial carcinoma are quite different. A subgroup analysis of ovarian cancer for combined 1-year, 3-year, and 5-year OS rates revealed that high plasma D-dimer level remains to be a risk factor for ovarian cancer patient (OR= 4.03, 2.34, 3.77, respectively, Figure 5). We did not conduct subgroup analysis in cervical and endometrial cancers since the included studies are too few.

## DISCUSSION

Plasma D-dimer, which is produced by the degradation of fibrin, has been widely recognized as a biomarker of hemostasis and fibrinolysis activation. It is playing an indispensable role in the determination of initial anticoagulant therapy in clinical practice. Recently, the elevation of plasma D-dimer level is observed in numeral clinical settings, including pregnancy, cardiovascular disease, cancer, post-surgery or trauma [17]. High D-dimer (DD) levels are report as a poor prognostic factor in patients with lung, prostate, cervical, and colorectal cancer. Recently, a large prospective study from the Vienna Cancer and Thrombosis study with 1178 cancer patients indicates that D-dimer associated with poor survival in solid cancer patients [18]. All the results demonstrated that D-dimer may play an important role in the tumorigenesis and prognosis.

In this study, we, for the first time, evaluated the prognostic significance of plasma D-dimer level in gynecological cancers. Patients with a high plasma D-dimer level have a worse 5-year prognosis than those with a low plasma D-dimer level (OR 4.12, 95% CI 3.04-5.58, P<0.00001). We also demonstrated that high plasma D-dimer level is a risk factor of prognosis in gynecological cancers by calculating the pooled HR of high plasma D-dimer (HR=2.09 95% CI 1.59-2.74, P<0.00001). We believe that this meta-analysis help definite the prognostic significance of plasma D-dimer level in gynecological tumors.

In terms of the mechanism by which D-dimer affect prognosis, classically, people believe that it is through the formation of VTE. H high D-dimer levels is found predictive of occurrence of VTE in patients with cancer [19]. VTE is a frequent complication of cancer, especially ovarian cancer (1.2%), brain tumors (1.2%), and pancreatic cancer (1.1%), and ranks major causes of death in cancer patients [20]. However, recently large scaled prospective studies found that the association of high D-dimer levels with poor prognosis was independent of VTE in hematologic malignancies and solid tumors [18]. It raised the question that D-dimer might be able to affect prognosis through a VTE independent pathway. It has been suggested that the application of anticoagulation medicine, such as the low molecular weight heparins, unfractionated heparin and vitamin K antagonists, exerts an encouraging effect on survival of cancer patients [21]. In this respect, it is of great interest that to investigate the use of anticoagulation reagents in cancer patients with elevated D-dimer levels. However, further studies are needed to figure out the particular biological mechanism of D-dimer in cancer patients.

There are some limitations concerning this study. First, the number of included studies is not limited and all came from Asia, indicating the existence of ethnicity bias. In addition, the cutoff value to define high and low D-dimer levels in seven included studies vary, which increased the difficulty of doing a pooled study. We believe that a standard and well-defined testing system of plasma D-dimer is necessary in clinical practice. Last but not least, although no obvious publication bias was revealed in this study, potential bias might still existed since positive results were more likely to be published.

In a nutshell, an elevated plasma D-dimer level is significantly associated with poor response in gynecological cancer patients. The plasma D-dimer level could be a prognostic biomarker of gynecological cancer patients.

## MATERIALS AND METHODS

### Literature search

Two investigators (Lei Xu and Bei Gao) independently searched eligible manuscripts in PubMed, Web of Science and China National Knowledge Infrastructure dating up to August, 2016. The following keywords and their combinations are applied in searching: “D dimer” or “D-dimer,” and “ovarian cancer” or “cervical cancer” or “endometrial cancer” or “Endometrial carcinomas,” and“prognosis” or “survival” from 2007–2016. The last search was performed on Sep 6, 2016. All of the eligible manuscripts and their references are retrieved for data extraction and analysis.

### Selection criteria

Studies met the following criteria were collected in this meta-analysis: (1) all gynecological cancers, including ovarian cancer, cervical cancer and endometrial cancer were all histopathological diagnosed; (2) the level of D-dimer were detected prior to surgery; (3) OS, DFS and hazard ratios (HRs) with their 95% confidence interval (95% CI) of OS/DFS of patients were evaluated in all studies; (4) if overlapping data were published in different reports by the same investigator, only the most complete one was included.

### Data extraction and quality assessment

Two independent investigators (Fan He and Bei Gao) assess the quality of these publications using tools in Revman5.3 recommended by Cochrane Collaboration. It features five different bias, including selection, performance, detection, attrition, reporting and other potential sources of bias. Each category is ranked low risk, high risk or unclear For each study, following information were extracted: author, year of publication, country, number of patients, plasma D-dimer detection method, cutting off value of high D-dimer level, OS, DFS, 60 months survival rates and HR with its 95% CI. If the data couldn't be obtained directly, we calculated from Kaplan-Meier survival curve. Kaplan-Meier survival curves were imported into digitizing software (Engauge Digitizer 4.1, Free Software Foundation) to extract for 1-year and 3-year survival rates. Two group members (Lei Xu and Huini Wu) extract data independently to prevent typos and mistakes and disagreements are resolved by discussion to reach consensus.

### Statistical analysis

All analysis were performed with Revman5.3 (The Nordic Cochrane Centre, the Cochrane Collaboration, Copenhagen, Denmark). Statistical heterogeneity between studies was determined by Cochran's Q test and Higgins I square. Heterogeneity was defined as P < 0.1 or I^2^ > 50%. In the absence of statistically significant heterogeneity, a fixed effects model is used to combine the data. Otherwise, a random effects model is applied. Publication bias is assessed by funnel plot with Revman5.3. Subgroup analysis is conducted by stratifying on tumor type. For all analyses, a two-sided P value less than 0.05 was considered statistically significant.
